# Supplementary material for: Blood Transcriptomes of Anti-SARS-CoV-2 Antibody-Positive Healthy Individuals Who Experienced Asymptomatic Versus Clinical Infection
Source: Front Immunol. 2021 Oct 5;12:746203. doi: 10.3389/fimmu.2021.746203 (PMC8523987; doi:10.3389/fimmu.2021.746203)
Supplement: Supplementary file 1 [file DataSheet_1.pdf]

Supplementary Material

Supplementary Table S1

Sequence of primers used for SYBR Green qPCR analysis

Gene	Forward primer sequence (5'->3')	Reverse primer sequence (5'->3')
GAPDH	CAACGGATTTGGTCGTATT	GATGGCAACAATATCCACTT
CLEC12A	ATGCCAGCCTGTTGAAGATAA	CAGGAGATAATCCCAGCCAATAG
GZMH	CAGCAGAGCCACTGAGATTT	TACCTTGGGCTACGTCCTTA
RSAD2	TGGTGAGGTTCTGCAAAGTAG	GTCACAGGAGATAGCGAGAATG
IFIT3	CTGCTGTGCAACATGGTTTAG	TCGGATACATTCTGTGGTTGG
PI3	AAAGGCCGTGTTCCATTCA	GCTCTTGCGCTTTGACTTTATC

Supplementary Table S2

Information on genes with decreased expression in healthy individuals with prior asymptomatic infection relatively to those with prior clinical SARS-CoV-2 infection

Gene	Description and Biological Terms Related (if applicable)
**IFIT3**	Interferon Induced Protein With Tetratricopeptide Repeats 3<br><br>Pathways: Innate Immune System and Interferon gamma signaling.<br>Terms: identical protein binding.
**IFI44L**	Interferon Induced Protein 44 Like<br><br>Diseases associated: Lymph Node Tuberculosis and Immunodeficiency 38 With Basal Ganglia Calcification.
**FOLR3**	Folate Receptor Gamma<br><br>Pathways: Innate Immune System and Endocytosis.<br>Terms: folic acid binding.
**PI3**	Peptidase Inhibitor 3.<br><br>Pathways: Innate Immune System and Developmental Biology.<br>Terms: serine-type endopeptidase inhibitor activity and peptidase inhibitor activity.
**RSAD2**	Radical S-Adenosyl Methionine Domain Containing 2 (Viperin)<br><br>Pathways: Innate Immune System and Interferon gamma signaling.<br>Terms: self-association and iron-sulfur cluster binding.
**ALOX15**	Arachidonate 15-Lipoxygenase<br>The enzyme acts on various polyunsaturated fatty acid substrates to generate various bioactive lipid mediators such as eicosanoids, hepoxilins, lipoxins, and other molecules. The encoded enzyme and its reaction products have been shown to regulate inflammation and immunity. [RefSeq, Aug 2017]<br><br>Pathways: Interleukin-4 and 13 signaling and Arachidonic acid metabolism.<br>Terms: iron ion binding and oxidoreductase activity, acting on single donors with incorporation of molecular oxygen, incorporation of two atoms of oxygen.

Supplementary Table S3

Information on genes with increased expression in healthy individuals with prior asymptomatic infection relatively to those with prior clinical SARS-CoV-2 infection

Gene	Description and Biological Terms Related (if applicable)
**DUSP8**	Dual Specificity Phosphatase 8<br><br>Pathways: Signaling by GPCR and MAPK signaling pathway<br>Terms: phosphatase activity and protein tyrosine/serine/threonine phosphatase activity
**CAC-NA2D2**	Calcium Voltage-Gated Channel Auxiliary Subunit Alpha2delta 2<br><br>Pathways: ERK Signaling and Activation of cAMP-Dependent PKA.<br>Terms: voltage-gated calcium channel activity and calcium channel regulator activity
**GCAT**	Glycine C-Acetyltransferase<br><br>Pathways: Glycine, serine and threonine metabolism, Viral mRNA Translation.<br>Terms: pyridoxal phosphate binding and glycine C-acetyltransferase activity.
**GZMH**	GranezymeH<br>Reported to be constitutively expressed in the NK (natural killer) cells of the immune system and may play a role in the cytotoxic arm of the innate immune response by inducing target cell death and by directly cleaving substrates in pathogen-infected cells.<br><br>Pathways are Peptide hormone metabolism and Metabolism of proteins.<br>Terms: serine-type endopeptidase activity.
**CLEC12A (MICL)**	C-lectine-like receptor:<br>Pathways are Innate Immune System and C-type lectin receptor signaling pathway.<br>Terms: include carbohydrate binding.
**CLEC1B (CLEC2)**	C-Type Lectin Domain Family 1 Member B<br>Natural killer (NK) cells express multiple calcium-dependent (C-type) lectin-like receptors, such as CD94 (KLRD1; MIM 602894) and NKG2D (KLRC4; MIM 602893), that interact with major histo-compatibility complex class I molecules and either inhibit or activate cytotoxicity and cytokine secre-tion. CLEC2 is a C-type lectin-like receptor expressed in myeloid cells and NK cell (Colonna et al., 2000 [PubMed 10671229])<br><br>Pathways: Response to elevated platelet cytosolic Ca2+ and C-type lectin receptor signaling pathway.<br>Terms: transmembrane signaling receptor activity and carbohydrate binding.
**BEX5**	Brain expressed X-linked5, Nerve Growth Factor Receptor-Associated Protein 2
**ENC1**	Ectodermal-Neural Cortex 1<br><br>Pathways are WNT Signaling.<br>Terms: actin binding.
**CYP3A43**	Cytochrome P450 Family 3 Subfamily A Member 43<br><br>Pathways are Paroxetine Pathway, Pharmacokinetics and Drug metabolism - cytochrome P450.<br>Terms: iron ion binding and oxidoreductase activity, acting on paired donors, with incorporation or reduction of molecular oxygen

Supplementary Figure S1

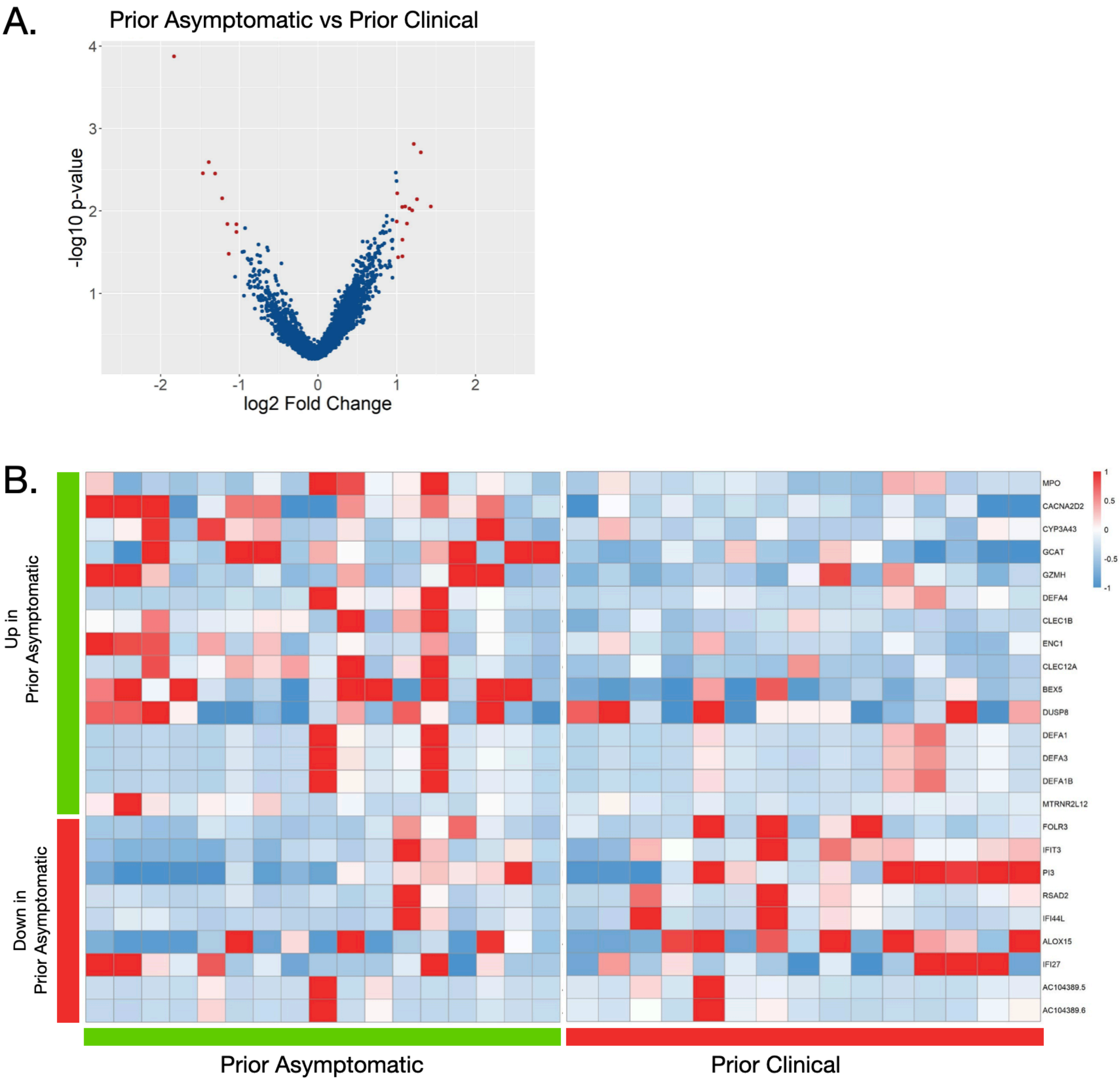

Supplementary Figure S1. Differential gene expression analysis in seropositive individuals with prior asymptomatic or clinical SARS-CoV-2 infection. (A) Volcano plot of all of differentially expressed genes of individuals with prior asymptomatic infection relatively to those with clinical ("symptomatic") SARS-CoV-2 infection. Significantly increased (right, 15 genes) or decreased(left, 9 genes) genes in are represented with red dots. The threshold for the adjusted p-value was set to 0.05 and for absolute log2(fold-change) to 1. (B) Heatmap of all differentially expressed genes in individuals with prior asymptomatic infection relatively to those with clinical ("symptomatic") SARS-CoV-2 infection, with raw expression values being scaled. The values for all samples (17 asymptomatic on the left and 15 clinical on the right) is plotted. The first 15 genes are increased in the Asymptomatic group, while the next 9 are decreased.

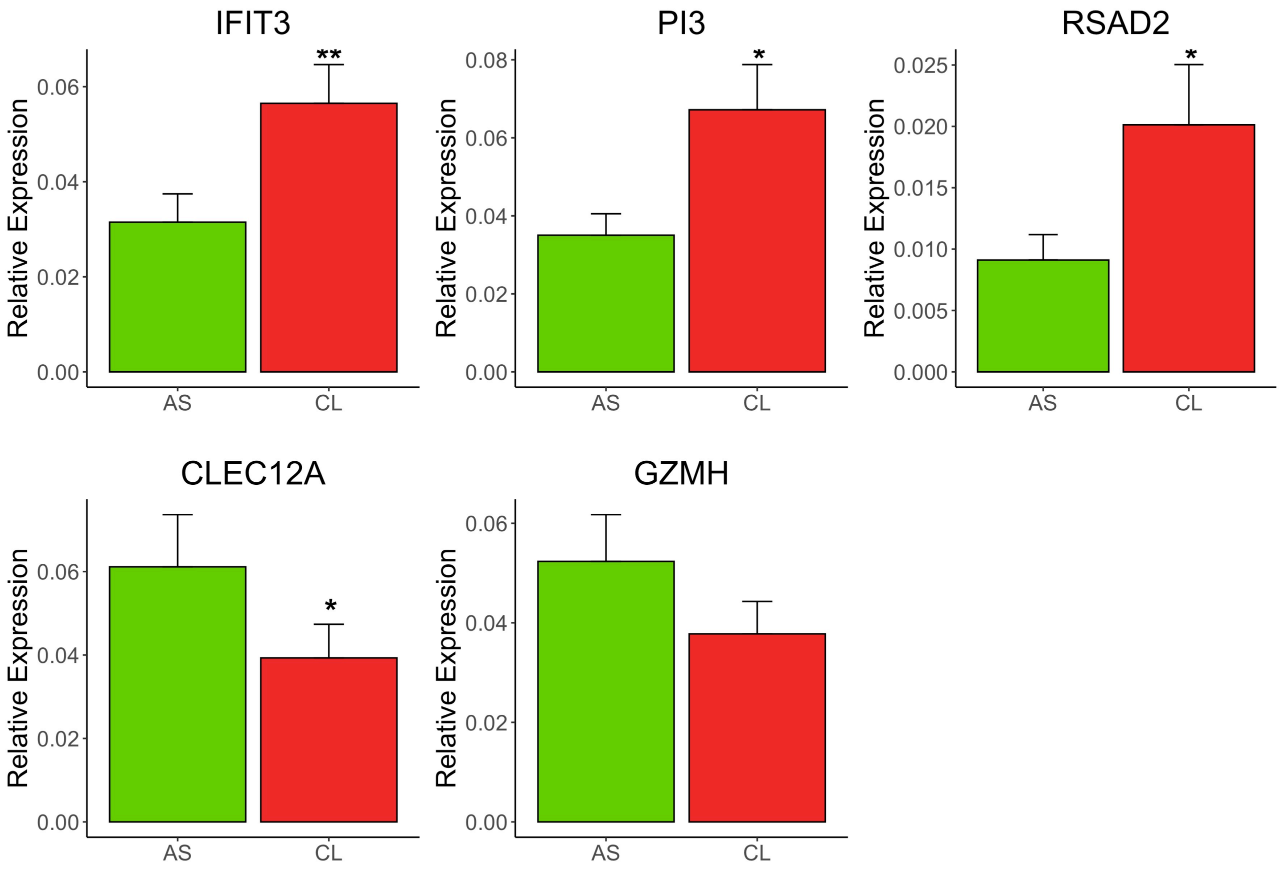

Supplementary Figure S2: PCR validation of differentially expressed genes in seropositive individuals with prior asymptomatic (AS, n=21) or clinical (CL, n=18) SARS-CoV-2 infection. The mRNA expression of 5 selected, differentially expressed genes according to RNA-seq) (3 and 2 decreased or increased, respectively, in prior asymptomatic infection relatively to clinical SARS-CoV-2 infection) was quantified with SYBR Green qPCR in cDNA derived from whole blood. *P<0.05, **P<0.01 by Mann-Whitney U test. Bars represent mean and standard error mean.
